# Role of Nitric Oxide Isoforms in Vascular and Alveolar Development and Lung Injury in Vascular Endothelial Growth Factor Overexpressing Neonatal Mice Lungs

**DOI:** 10.1371/journal.pone.0147588

**Published:** 2016-01-22

**Authors:** Mansoor A. Syed, Rayman Choo-Wing, Robert J. Homer, Vineet Bhandari

**Affiliations:** 1 Division of Perinatal Medicine, Department of Pediatrics, Yale University School of Medicine, 333 Cedar Street, New Haven, CT 06520–8064, United States of America; 2 Department of Pathology, Yale University School of Medicine, 310 Cedar Street, New Haven, CT 06520, United States of America; Goethe University, GERMANY

## Abstract

**Background:**

The role of vascular endothelial growth factor (VEGF)-induced 3 different nitric oxide synthase (NOS) isoforms in lung development and injury in the newborn (NB) lung are not known. We hypothesized that VEGF-induced specific NOS pathways are critical regulators of lung development and injury.

**Methodology:**

We studied NB wild type (WT), lung epithelial cell-targeted VEGF165 doxycycline-inducible overexpressing transgenic (VEGFTG), VEGFTG treated with a NOS1 inhibitor (L-NIO), VEGFTG x NOS2^-/-^ and VEGFTG x NOS3^+/-^ mice in room air (RA) for 7 postnatal (PN) days. Lung morphometry (chord length), vascular markers (Ang1, Ang2, Notch2, vWF, CD31 and VE-cadherin), cell proliferation (Ki67), vascular permeability, injury and oxidative stress markers (hemosiderin, nitrotyrosine and 8-OHdG) were evaluated.

**Results:**

VEGF overexpression in RA led to increased chord length and vascular markers at PN7, which were significantly decreased to control values in VEGFTG x NOS2^−/−^ and VEGFTG x NOS3^+/-^ lungs. However, we found no noticeable effect on chord length and vascular markers in the VEGFTG / NOS1 inhibited group. In the NB VEGFTG mouse model, we found VEGF-induced vascular permeability in the NB murine lung was partially dependent on NOS2 and NOS3-signaling pathways. In addition, the inhibition of NOS2 and NOS3 resulted in a significant decrease in VEGF-induced hemosiderin, nitrotyrosine- and 8-OHdG positive cells at PN7. NOS1 inhibition had no significant effect.

**Conclusion:**

Our data showed that the complete absence of NOS2 and partial deficiency of NOS3 confers protection against VEGF-induced pathologic lung vascular and alveolar developmental changes, as well as injury markers. Inhibition of NOS1 does not have any modulating role on VEGF-induced changes in the NB lung. Overall, our data suggests that there is a significant differential regulation in the NOS-mediated effects of VEGF overexpression in the developing mouse lung.

## Introduction

It is well known that increased vascular endothelial growth factor (VEGF) induces multiple effects in the adult lung including inflammation, parenchymal and vascular remodeling, edema, mucus metaplasia, myocyte hyperplasia and airway hyper-responsiveness [1]. Nitric oxide (NO) has been shown to be critical for downstream signaling of most of the above noted VEGF-induced effects [2]. NO production is mostly controlled by 3 isoforms of the NO synthase (NOS) enzymes–NOS1 (neuronal or nNOS), NOS2 (inducible or iNOS) and NOS3 (endothelial or eNOS). It appears that most of VEGF-induced NOS-mediated effects occur via NOS2 and NOS3, as NOS1 was not found to be increased in the adult lung [2].

In the developing lung, VEGF has been shown to be critical for vascular as well as parenchymal maturation, including surfactant production [3, 4]. Interestingly, in contrast to the adult lung, it has been shown that increased VEGF enhances the expression of all 3 NOS isoforms in the newborn (NB) lung [4]. Furthermore, while VEGF-induced pulmonary hemosiderosis and endothelial permeability was NO-dependent, the VEGF-induced pulmonary maturational effects, including surfactant production in the NB lung, was NO-independent [4].

Given the critical role of VEGF-induced NO-mediated effects in vascular development in the NB lungs, we hypothesized that the 3 NOS isoforms would differentially regulate vascular markers in the VEGF-induced alterations in the NB lung. Our aims were to study the impact of increased VEGF exposure to the developing lung on lung morphometry, cell proliferation, vascular markers, vascular permeability, injury, oxidative stress markers and surfactant proteins, with the absence/inhibition of NOS 1 to 3.

We show that VEGF exposure leads to increased alveolar size (based on chord length), which is reversed by NOS2/3 absence, but not by NOS1 inhibition. VEGF induction led to decreased cell proliferation (based on Ki67 staining), which was reversed by NOS2/3 absence, but not by NOS1 inhibition. VEGF exposure led to a significant induction of vascular markers, as evidenced by increased von Willebrand factor (vWF; a marker for endothelial cells), CD31 (a marker for an endothelial cell adhesion molecule), VE-cadherin (an adhesion molecule located at the junctions between endothelial cells), collagen IV (a marker for the basal lamina of endothelial cells), and Angiopoietin 2 (Ang2; produced and stored in endothelial/epithelial cells) but suppression of Angiopoietin 1 (Ang1; produced by vascular support cells) and Notch2 (a transmembrane receptor mostly found in the pulmonary endothelium). VEGF induction with inhibition of NOS1 led to no change in collagen IV, VE-cadherin, Ang2, Notch2, vWF and CD31 protein expression. VEGF induction in the absence of NOS2 and NOS3 led to a significant reduction in the expression of vWF, CD31, VE-cadherin, and Ang2 with not much change in collagen IV. In contrast, there was an increased expression of Ang1 and Notch2 proteins.

Activation of VEGF led to increased vascular permeability based on increased bronchoalveolar lavage (BAL) protein content and decreased claudin 1 (endothelial cell tight junction marker) expression, which was significantly decreased (but not to normal levels) and expression levels restored, respectively, in NOS2/3 absence, but not by NOS1 inhibition. In addition, the inhibition of NOS2 and NOS3 resulted in a significant decrease in VEGF-induced hemosiderin, nitrotyrosine- and 8-OHdG positive cells. NOS1 inhibition also led to a decrease in VEGF-induced hemosiderin, but had no significant effect on the other 2 injury markers.

We confirmed that while VEGF induction led to increased expression of surfactant proteins B and C (SP-B and -C), with no impact on SP-A and -D, there was no impact of the concomitant absence/inhibition of NOS 1 to 3 on these effects.

Finally, we noted a significant increase in alveolar size upon VEGF-induction in room air (from postnatal or PN day 5–14), after exposure to hyperoxia from PN1-4 (the mouse broncho-pulmonary dysplasia or BPD model [5, 6]), suggesting a detrimental response to VEGF treatment.

## Materials and Methods

### Animals

Dual transgenic CC10-rtTA-*VEGF* (*VEGF*-TG) mice overexpressing the human isoform, VEGF_165_, a kind gift from Jack Elias, MD, were generated and characterized as previously described [1, 2]. Male *VEGF*-TG heterozygote mice were bred with female *NOS2*^−/−^ and *NOS3*^−/−^ mice (both on C57BL/6J background, as previously described [2]) to generate the *VEGF*-TG/ *NOS2*^−/−^ and *VEGF*-TG/ *NOS3*^−/−^ mice colonies. However, due to high perinatal mortality, we could not obtain sufficient number of *VEGF*-TG/ *NOS3*^−/−^ homozygous neonatal mice for replicative experiments; hence, we utilized *VEGF*-TG/ *NOS3*^+/−^ heterozygous for our studies. The *VEGF*-TG mice express VEGF_165_ in the lung with maternal exposure to doxycycline (dox) in the drinking water, leading to trans-mammary activation in the TG (+) pups [4].

All animal work was approved by the Institutional Animal Care and Use Committee at the Yale University School of Medicine.

### Experimental Design

Two independent models were used for our studies, with a minimum of 5 animals in each experimental and control group, as appropriate.

1. *VEGF*-TG (+) postnatal day 7 (PN7) room air (RA) model: To evaluate the effects of VEGF-165 overexpression on lung development, we activated VEGF overexpression in *VEGF*-TG (+) mice from PN1-PN7 by administration of dox in the maternal drinking water, in RA. NB WT mice with maternal administration of dox water served as a control. We used this experimental model with *VEGF*-TG/ *NOS2*^−/−^ and *VEGF*-TG/ *NOS3*^+/−^ groups to ascertain the impact of these specific NO-isoforms. For NOS1 inhibition, we used the specific inhibitor Vinyl-L-NIO hydrocholride (L-NIO; Santa Cruz Biotechnology) in the dose 10 mg/kg for 7 days. The animals were killed on PN7 and lung tissue was harvested for analysis.
2. NB mouse hyperoxia-induced BPD model: For the NB animals, exposure to hyperoxia (along with their mothers) was performed by placing mice in cages in an airtight Plexiglass chamber (55 × 40 × 50 cm), as described previously [5]. Exposure to 100% oxygen was initiated on PN1 and continued until PN4 (saccular stage of mouse lung development). On PN5, all animals were placed in RA up to PN14 to allow for a period (alveolar stage of mouse lung development) of recovery. Two lactating dams were used and alternated in hyperoxia and RA every 24 h, during the hyperoxia phase (PN1-4) of the experimental model. Using this experimental model, NB wild-type (WT) mouse lungs at PN14 have the phenotype mimicking human BPD, as has been reported previously by us [5, 7] and other investigators [6]. For the *VEGF*-TG (+) animals in this model, we activated VEGF overexpression from PN5-PN14 (RA recovery phase) by administration of dox in the maternal drinking water. Mice were sacrificed on PN14.

### Histology

Lung tissues were obtained from NB mice from the RA (PN7) and BPD (PN14) models as well as the VEGF/NOS groups. They were subjected to a standard protocol for lung inflation (25 cm) and fixed overnight in 10% buffered formalin [5]. After washing in fresh PBS, fixed tissues were dehydrated, cleared, and embedded in paraffin by routine methods. Sections (5 μm) were collected on Superfrost Plus positively charged microscope slides (Fisher Scientific Co., Houston, Texas, USA), deparaffinized, and stained with hematoxylin & eosin, as described previously [8].

### Lung Morphometry

Five-micrometer lung sections were stained with hematoxylin and eosin. Each slide contained tissue from the left lobe and represented an individual animal. Five randomly chosen areas from each section were photographed with the ×10 objective. Alveolar size was estimated from the mean chord length of the airspace, as described previously [9]. Chord length increases with alveolar enlargement. Alveolar septal wall thickness was estimated using Image J software, adapting the method described previously for bone trabecular thickness, for the lung [10].

### Quantitative Real-Time PCR Analysis

Lungs were homogenized, and total RNA was isolated with TRIZOL (Invitrogen, Carlsbad, CA) and Qiagen RNeasy kit (Qiagen). First-strand cDNA was synthesized with iScript cDNA Synthesis kit for Real-Time-PCR (Bio-Rad, Hercules, CA) according to the manufacturer’s instructions. Real-time PCR reaction was performed in a 20-μL volume with SYBR Green (Bio-Rad, Hercules, CA) with the use of pooled cDNA samples.

Surfactant proteins: (SP)-A, 5′-TCTTGACTGTTGTTGCTGGC-3′, 5′-AGAAGCCCCATCCAGGTAGT-3′; SP-B, 5′-GACCTGTGCCAAGAGTGTGA-3′, 5′-GGCATAGCCTGTTCACTGGT-3′; SP-C, 5′-GCAAAGAGGTCCTGATGGAG-3′, 5′-GCCCGTAGGAGAGACACCTT-3′; SP-D, 5′-CTCTCGCAGAGATCAGTACC-3′, 5′-GGAAAGCAGCCTTGTTGTGG-3

### Bronchoalveolar lavage (BAL)

BAL was performed in PN7 neonatal mice and hVEGF levels measured by ELISA (R&D), as previously described [4]. Total protein content in the BAL was quantified, as per manufacturer’s (Bio-Rad) instructions.

### Western Blot

Ang1, Ang2 (both from Millipore), Notch 2, VE-cadherin, Claudin 1 (all from Cell Signaling), Collagen IV (Abcam), SP-A, SP-B, SP-C, SP-D, Claudin-1, NOS1, NOS2, NOS3, β-actin (Santacruz) antibodies were used for western blotting.

### Immunohistochemistry

Immunohistochemistry was performed on formalin-fixed, paraffin-embedded tissue sections as previously described [5, 8, 11]. All primary antibody incubations were performed overnight at 4°C. The primary antibodies were used at the following concentrations: NOS1, NOS2, NOS3 (1:400), hemosiderin, nitrotyrosine, 8-OHdG (1:500), anti-vWF 1:200 (Abcam, Cambridge, MA); Ki-67 (Novus), 1:200; CD31 (Dako, CA), 1:50. Antigen retrieval was performed for CD31 and Ki-67 with citrate buffer (Dako, CA). After immunostaining of mouse sections, slides were coded, and six to eight images per sample were randomly selected and captured at ×200 or ×400 magnification for quantification of Ki-67, vWF and CD31-expressing cells, respectively, by a blinded investigator (MS). Quantification of Ki67, CD-31 and vWF were performed using Image J software (NIH).

### Statistical Analyses

Values are expressed as mean ± SEM. Groups were compared with the 2-way ANOVA, corrected for multiple comparisons by the Tukey test, using GraphPad Prism 3.0 (GraphPad Software, Inc., San Diego, CA), as appropriate. A p<0.05 was considered statistically significant.

## Results

In an effort to understand the VEGF-NO signaling in NB mice lungs in relation to vascular and alveolar development as well as lung injury–factors that have been considered critical both for pulmonary development and the pathogenesis of BPD–we focused our initial experimental studies on the contribution of the 3 NOSs. We have previously reported that all 3 NOSs are upregulated in our *VEGF*-TG (+) model and showed that there were NO-dependent and NO-independent effects based on pan-NOS inhibition [4]. We have also previously confirmed the specificity of the dox-induction of VEGF in our TG model, and hence, did not use regular water-exposed animals as additional controls [4]. In these series of experiments, we attempt to parse out the individual contribution of the NOSs to VEGF downstream signaling in terms of alveolar architecture, vascular development, cell proliferation, markers of lung injury, and surfactant protein expression at PN7, in RA. In addition, we have assessed the role of VEGF exposure during the RA recovery phase of a mouse BPD model.

### Effect of specific NOS isoform inhibition on morphometry and surfactant protein expression in the neonatal VEGF-TG (+) lung at PN7 in RA

**Fig 1:**
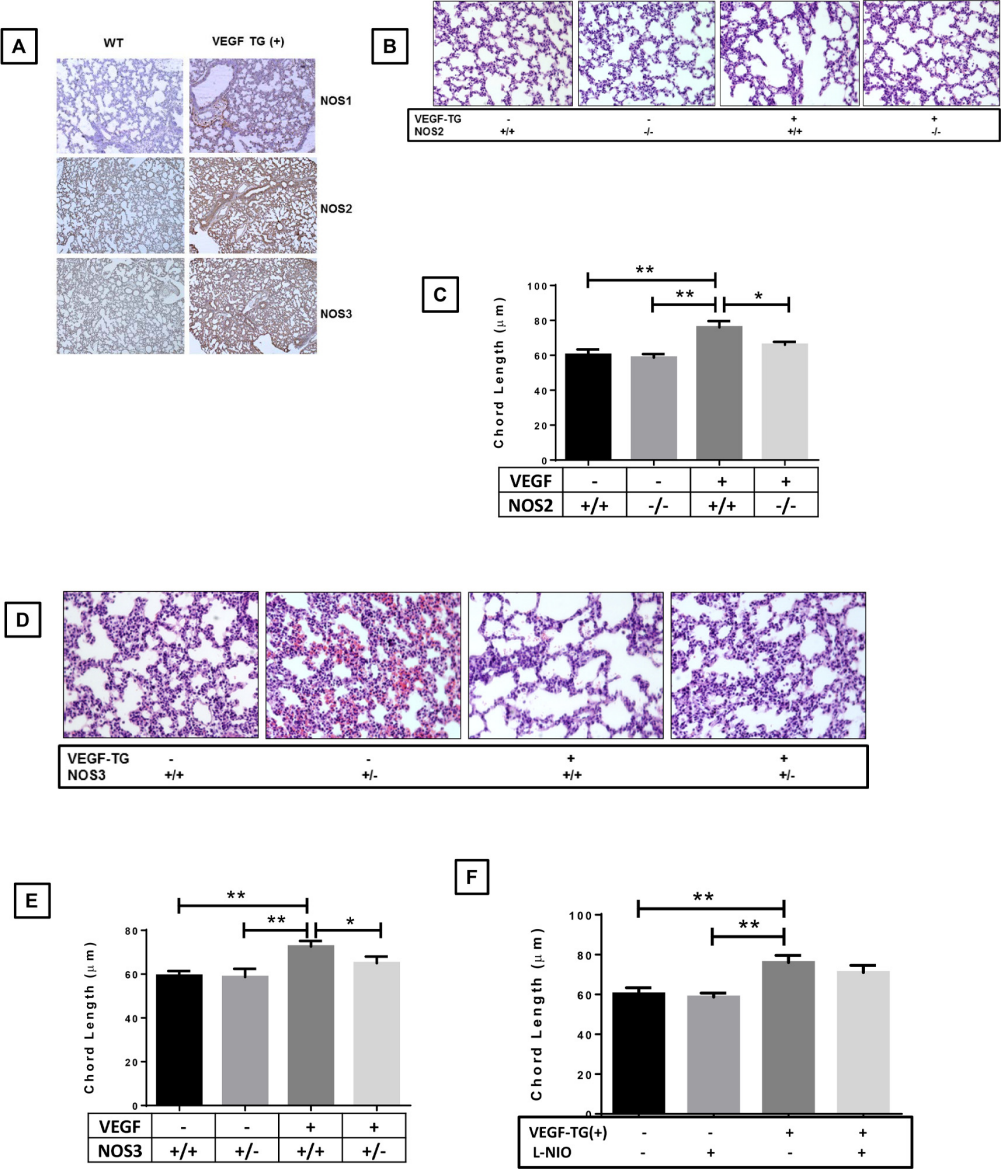
Role of NOS isoforms in VEGF overexpressed lung phenotype. VEGF TG+ and VEGF TG- mice were received DOX water from PN day1 to 7. (**A**) NB lungs from a VEGF TG+ showed increased NOS1, NOS2 and NOS3 staining of the endothelial and inflammatory cells was observed in the PN7 lung, compared with control lung samples. The figures are illustrative of a minimum of 5 animals in each group. (**B** and **C**) Alveolar size, as measured by chord length, confirmed features noted on lung histology in VEGF TG-, VEGF TG+, VEGF-TG/ NOS2^−/−^ (**D** and **E**) Similarly, chord length of VEGF TG-, VEGF TG+ and VEGF-TG/ NOS3^+/−^ and (**F**) chord lengths of VEGF TG+/LNIO groups. Each bar represents the mean ± SEM for a minimum of four animals, *P < 0.05 and **P ≤ 0.01, 2-way ANOVA followed by Tukey test. NOS: nitric oxide synthase; VEGF: vascular endothelial growth factor; TG-: transgene negative; TG+: transgene positive; DOX: doxycycline; NB: newborn; PN: postnatal.

VEGF is a strong and selective stimulator of all 3 NOS isoforms in the developing mouse lung as evaluated by us previously by mRNA expression [4] and confirmed by immunohistochemistry in the *VEGF*-TG (+) neonatal lung in RA (**Fig 1A**).

Next, we evaluated the effects of specific NOS1, NOS2 and NOS3 inhibition/deletion in VEGF induced changes in alveolar architecture in the PN7 neonatal lung. VEGF overexpression in RA led to increased chord length at PN7, which was significantly decreased to control values in *VEGF*-TG / *NOS2*^−/−^ and *VEGF*-TG / *NOS3*^+/−^ lungs (**Fig 1B–1E**). However, we found no noticeable effect on chord length in *VEGF*-TG / NOS1 inhibited group (**Fig 1F**).

We have previously reported that upregulation of surfactant proteins B and C secondary to VEGF on the developing neonatal lung is NO-independent [4]. NOS1 inhibited group had decreased expression of NOS1 **(S1 Fig)**, but did not show any noticeable changes in surfactant mRNA levels **(S2A Fig)**. We confirmed our observation utilizing the *VEGF*-TG / *NOS2*^−/−^ and *VEGF*-TG / *NOS3*^−/−^ mice lungs, at the mRNA and protein levels **(S2B–S2D Fig)**, too.

### Effect of specific NOS isoform inhibition on cell proliferation in the neonatal VEGF-TG (+) lung at PN7 in RA

**Fig 2:**
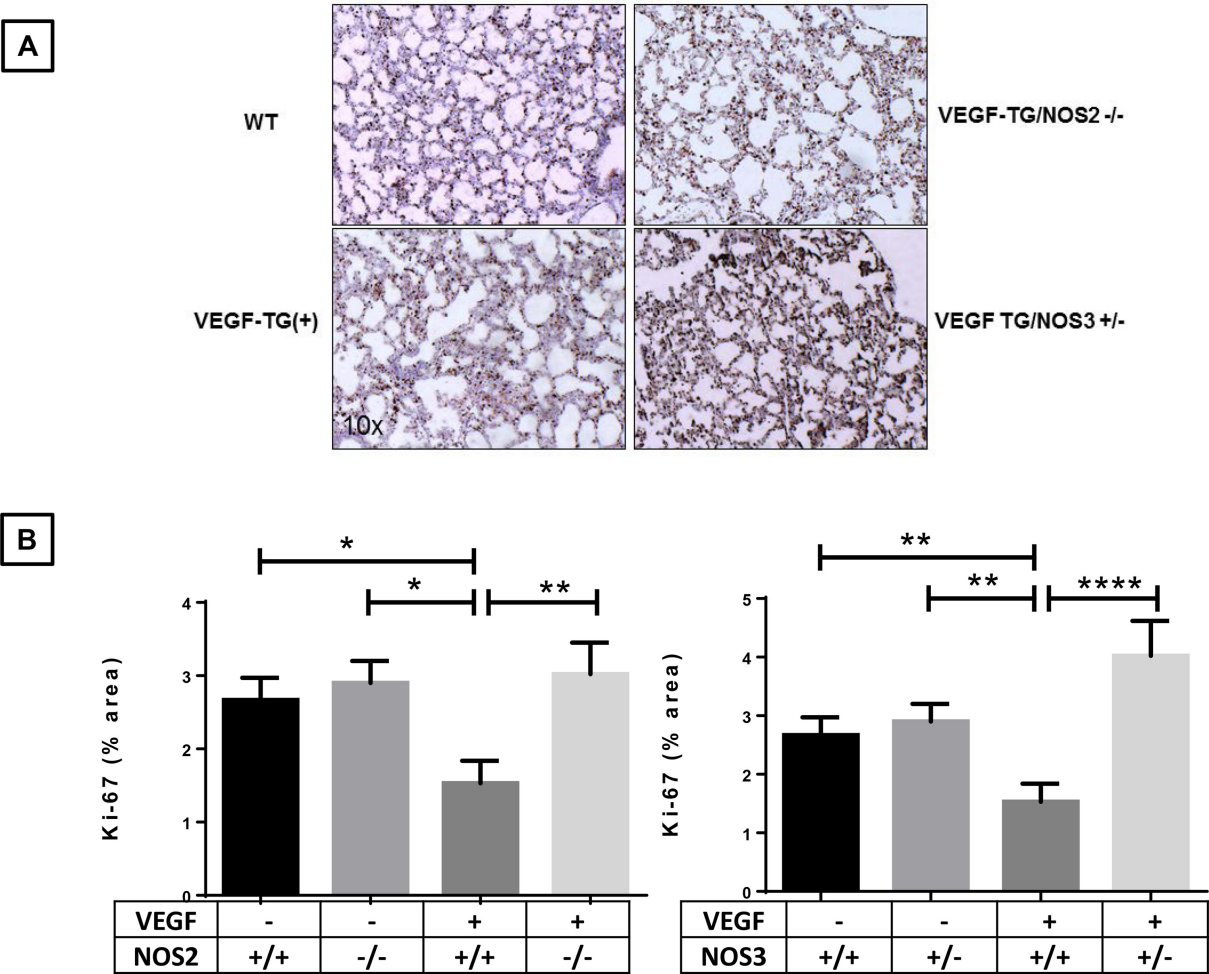
Role of NOS isoforms in VEGF overexpressed lung cell proliferation. (**A-C**) Representative examples of Ki67 (brown) staining in lungs from 7-day-old VEGF TG-, VEGF TG+, VEGF-TG/ NOS2^−/−^ and VEGF-TG/ NOS3^+/−^. All received DOX water from PN day1 to 7. Decreased percentage of Ki67 staining area in VEGF TG+, which is increased in VEGF-TG/ NOS2^−/−^ and VEGF-TG/ NOS3^+/−^ lungs. Each bar represents the mean ± SEM for a minimum of three animals. *P < 0.05, **P ≤ 0.01, and ****P ≤ 0.0001, 2-way ANOVA followed by Tukey test. NOS: nitric oxide synthase; VEGF: vascular endothelial growth factor; TG-: transgene negative; TG+: transgene positive; DOX: doxycycline; PN: postnatal.

To determine whether the observed differences in lung morphology in *VEGF*-TG (+), *VEGF*-TG / *NOS2*^−/−^ and *VEGF*-TG / *NOS3*^+/−^ may be due, at least in part, to different cell proliferation rates, we studied the pulmonary proliferative activity by immunohistochemical analysis using the proliferation marker Ki67. The pulmonary Ki67 quantification was significantly lower in *VEGF*-TG (+) animals compared with WT mice (**Fig 2A**). The proliferative activity of *VEGF*-TG / *NOS2*^−/−^ and *VEGF*-TG/ *NOS3*^+/−^ lungs were higher than in *VEGF*-TG (+) PN7 mice lung; however, *VEGF*-TG / *NOS2*^−/−^ mice lungs showed less staining than *VEGF*-TG / *NOS3*^+/−^ lungs (**Fig 2A**). Since we found no impact on the alveolar size in *VEGF*-TG / NOS1 inhibited group, we did not evaluate Ki67 in those mice lungs.

These data would suggest that restoration of the lung architecture upon NOS inhibition in the VEGF overexpressing neonatal lung could be driven, at least in part, by the increased cell proliferative capacity.

### Effect of specific NOS isoform inhibition on vascular development in the neonatal VEGF-TG (+) lung at PN7 in RA

**Fig 3:**
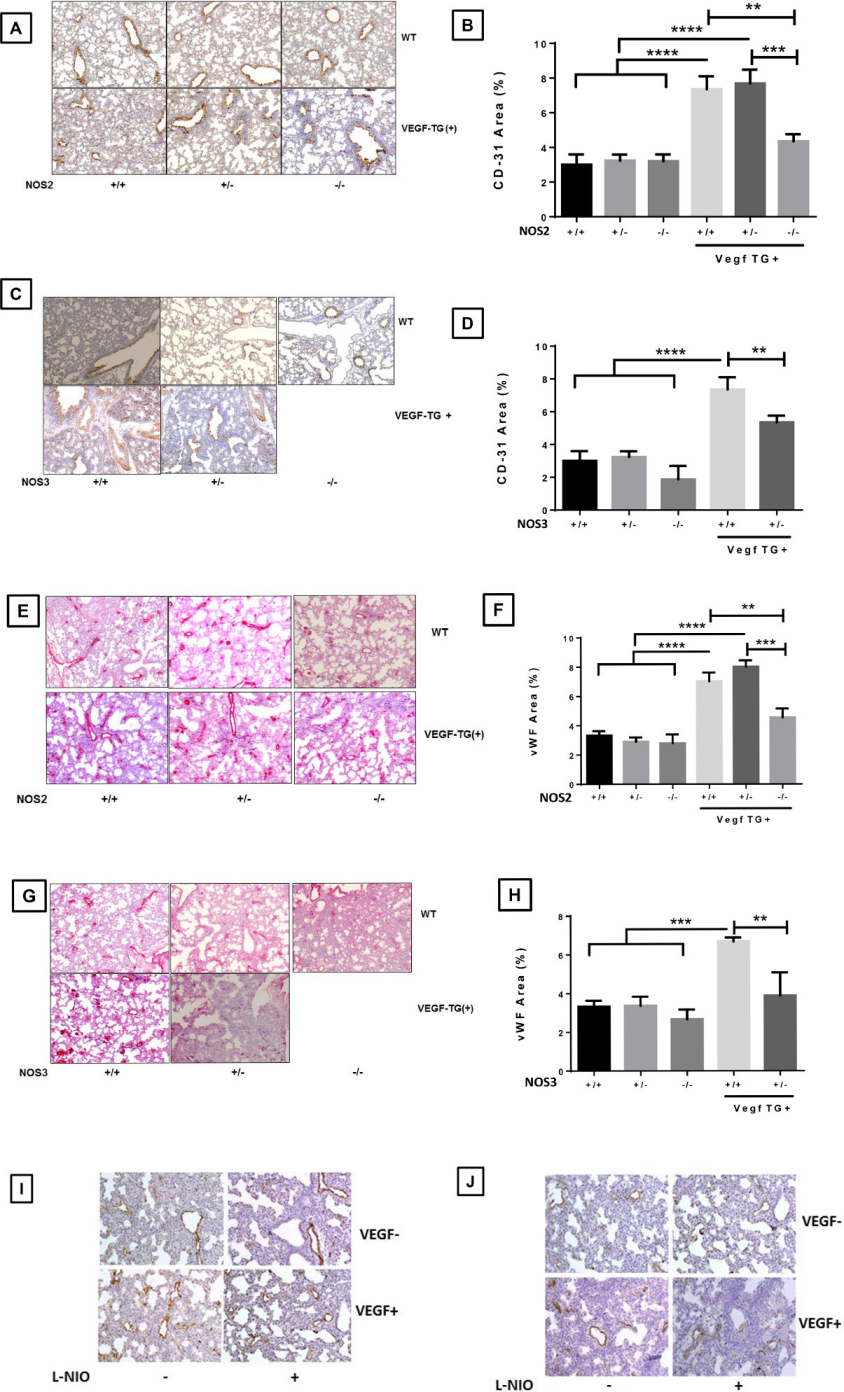
Role of NOS isoforms in VEGF overexpressed lung on the vascular markers CD31 and vWF. VEGF TG-, VEGF TG+, VEGF-TG/ NOS2^−/−^ and VEGF-TG/ NOS3^+/−^ mice exposed to DOX water from PN1-PN7. (**A** and **B**) Increased expression of lung CD31 in VEGF TG+ PN7 newborn mice, which was significantly decreased in VEGF-TG+/ NOS2^−/−^ and VEGF-TG/ NOS3^+/−^ (**C** and **D**) mice lungs. Similarly another vascular marker vWF showed decreased staining VEGF-TG+/ NOS2^−/−^ (**E** and **F**) and VEGF-TG/ NOS3^+/−^ (**G** and **H**) mice lungs as compared to VEGF TG+ lungs. (**I and J**) Increased expression of lung vWF and CD31 in VEGF TG+ PN7 newborn mice, with no significant change in L-NIO treated VEGF-TG+ animals. Each bar represents the mean ± SEM for a minimum of four animals. **P ≤ 0.01, ***P ≤ 0.001, ****P ≤ 0.0001, 2-way ANOVA followed by Tukey test. NOS: nitric oxide synthase; VEGF: vascular endothelial growth factor; TG-: transgene negative; TG+: transgene positive; DOX: doxycycline; PN: postnatal; vWF: von Willebrand factor.

We utilized CD31 and von Willbebrand Factor (vWF) as markers for lung vascular development. CD31 expression in WT, *NOS2*^−/−^ and *NOS3*^+/−^ mouse lungs exposed to DOX. As expected, *VEGF*-TG (+) lungs showed increased CD31 and vWF staining on immunohistochemistry, but this was markedly decreased in *VEGF*-TG / *NOS2*^−/−^ and *VEGF*-TG / *NOS3*^+/−^ lungs, confirmed by quantification (**Fig 3A–3H**). As with lung morphometry, we found no noticeable effect on CD31 and vWF expression in *VEGF*-TG / NOS1 inhibited group (**S2 Fig**).

### Effect of specific NOS isoform inhibition on angiogenic markers in the neonatal VEGF-TG (+) lung at PN7 in RA

**Fig 4:**
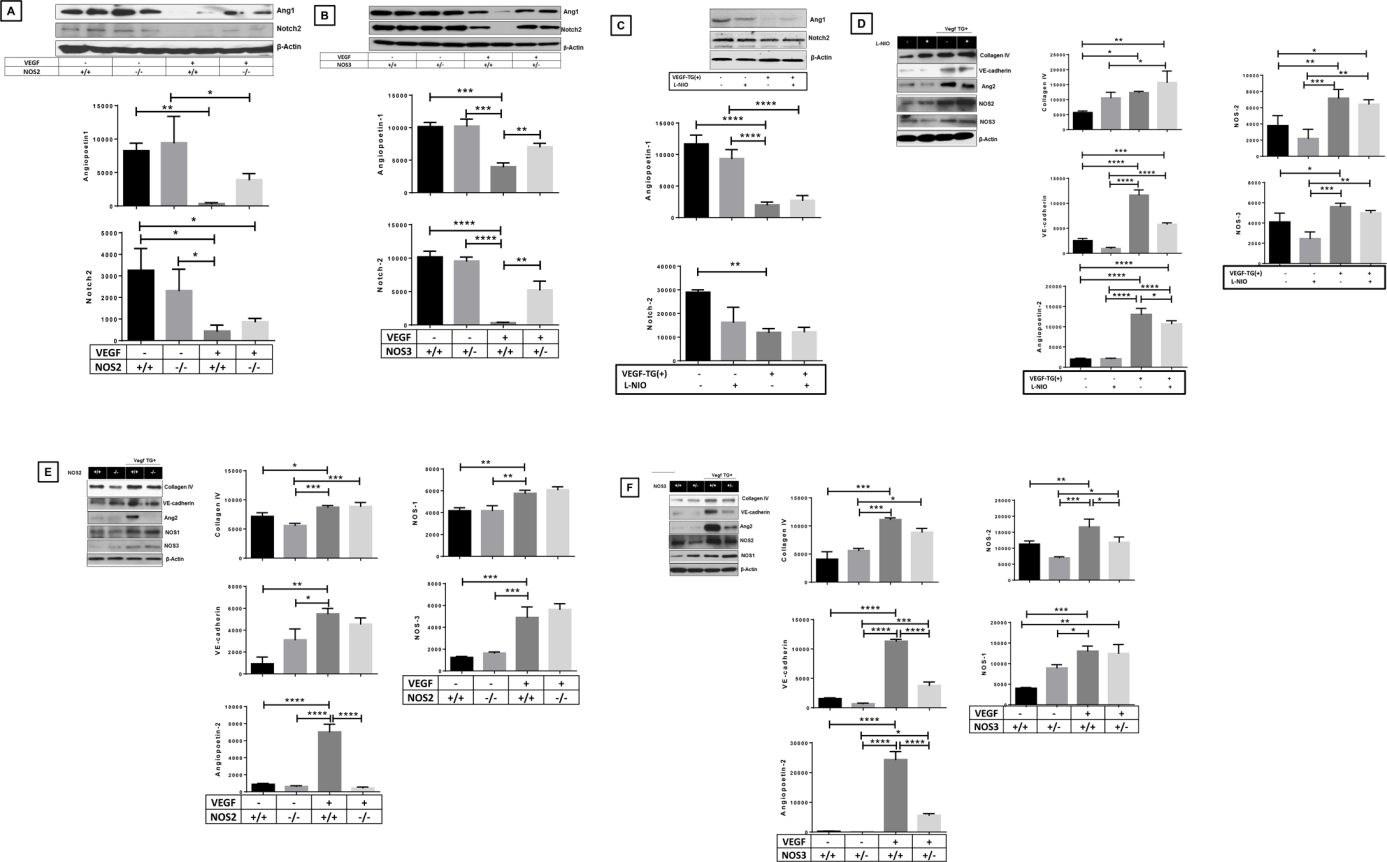
Role of NOS isoforms in VEGF overexpressed lung on angiogenic markers. NB VEGF TG-, VEGF TG+ and VEGF-TG/ NOS3^+/−^ mice were sacrificed at PN7. All received DOX water from PN day1 to 7. (**A** and **B**) Ang1 and NOTCH2 proteins, with β-actin as controls, were detected by western blotting and analyzed by densitometry. (**C**) Ang1 and NOTCH2 proteins, with β-actin were detected by western blotting (and densitometry) in L-NIO (NOS1 inhibitor) treated VEGF TG mice. (**D—F**) Protein levels (Western blot and densitometry) of Collagen IV, VE-cadherin, Ang2, NOS1, NOS2 and NOS3 are performed in indicated groups of PN7 mouse lungs. The figure is representative of n = 3 mice per group, *P < 0.05, **P ≤ 0.01, ***P ≤ 0.001 and ****P ≤ 0.0001, 2-way ANOVA followed by Tukey test. NOS: nitric oxide synthase; VEGF: vascular endothelial growth factor; NB: newborn; TG-: transgene negative; TG+: transgene positive; PN: postnatal; DOX: doxycycline; Ang: angiopoietin.

To determine whether specific NOS isoform inhibition impact on angiogenic markers, we evaluated the expression of angiopoietin 1 (Ang1) and Notch2 in the *VEGF*-TG (+) lung. Activation of the VEGF transgene caused decreased Ang1 and Notch2 expression, which reverted to control levels, in *VEGF*-TG/ *NOS2*^−/−^ and *VEGF*-TG/ *NOS3*^+/−^ lungs (**Fig 4A–4B**). No noticeable changes were noted with Ang1 and Notch2 expression in L-NIO treated VEGF-TG (**Fig 4C**). Western Blot analysis of Collagen IV, Ang2 and VE-cadherin also showed a similar pattern as with Ang1 and Notch2 in the different groups (**Fig 4D, 4E and 4F**).

### Effect of specific NOS isoform inhibition on lung edema and claudin 1 expression in the neonatal VEGF-TG (+) lung at PN7 in RA

**Fig 5:**
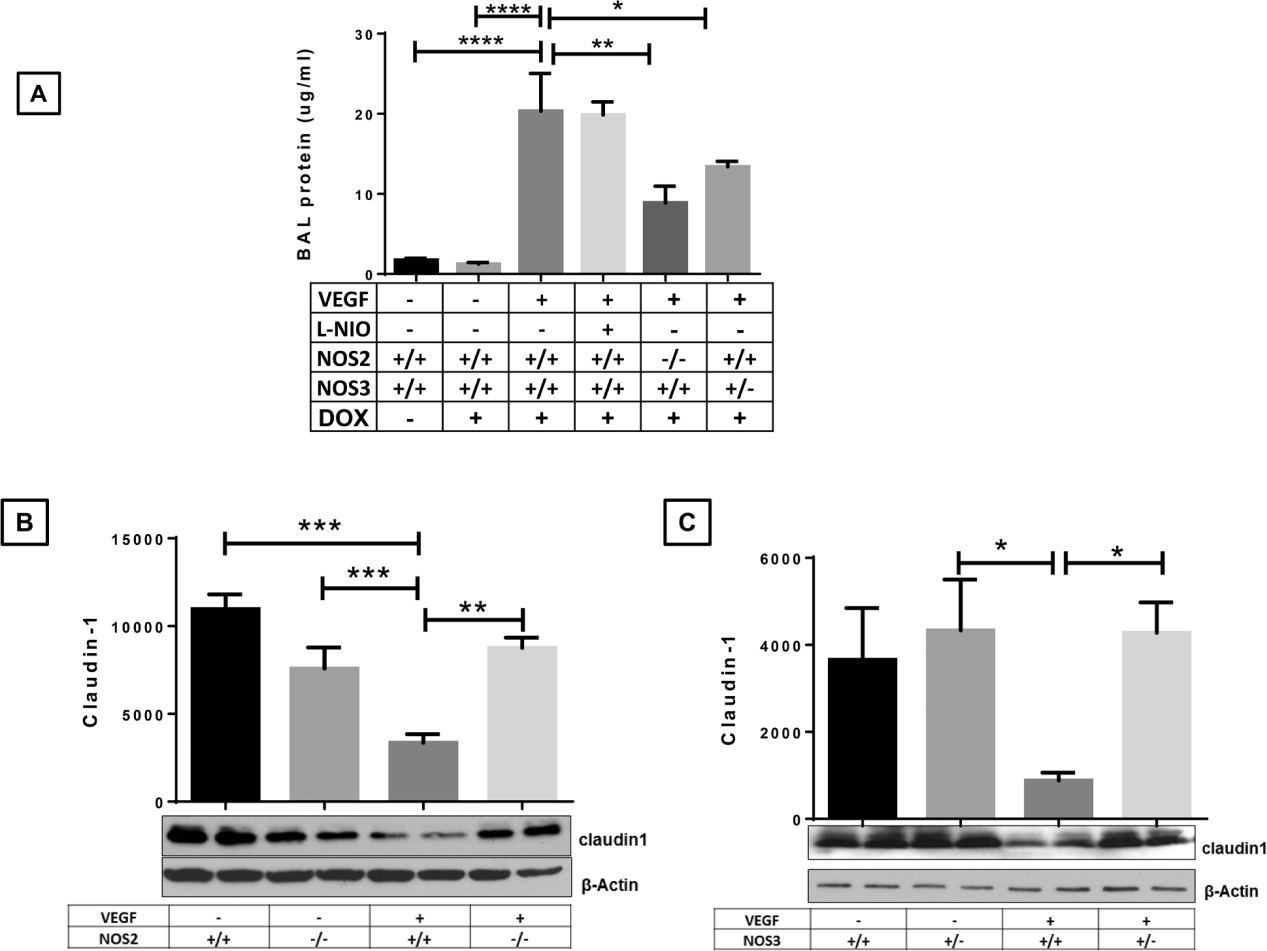
Role of NOS isoforms in VEGF overexpressed lung on BAL protein levels and endothelial barrier protein (claudin 1) expression. (**A**) BAL fluid was isolated from PN7 mouse lungs and protein content was measured. (**B** and **C**) Claudin 1 expression was decreased in PN7 VEGF TG+ mice lung as compared to control lungs. Inhibition of NOS2 and NOS3 in the presence of VEGF TG restored the claudin 1 protein expression levels. Each bar represents the mean ± SEM for a minimum of four animals. *P < 0.05, **P ≤ 0.01, ***P ≤ 0.001 and ****P ≤ 0.0001, 2-way ANOVA followed by Tukey test. NOS: nitric oxide synthase; VEGF: vascular endothelial growth factor; BAL: bronchoalveolar; TG+: transgene positive.

Pulmonary endothelial permeability plays major role in lung injury and claudin 1 is one of the major junctional proteins which enhance vascular integrity after injury [12, 13]. As noted (**Fig 5A**), transgenic VEGF caused a significant increase in NB lung BAL proteins. The NOS pathway played a significant role in this response because VEGF-induced increased NB lung BAL proteins level were decreased in *VEGF*-TG / *NOS2*^−/−^ and *VEGF*-TG / *NOS3*^+/−^, but not by NOS1 inhibition (**Fig 5A**). Thus, VEGF increases vascular permeability in the newborn murine lung via a mechanism(s) that is, at least partially, NOS2 and NOS3-dependent.

We hypothesized that increased edema (as evidenced by BAL protein levels) in PN7 *VEGF*-TG (+) lungs are due to involvement of the endothelial barrier proteins. Activation of VEGF TG down-regulates claudin 1 in *VEGF*-TG (+) lungs, with inhibition of NOS2 and NOS3 pathways restoring claudin 1 expression to the normal level (**Fig 5B and 5C**).

It is important to clarify that the vascular development markers are not a direct measure of microvessel numbers. We want to emphasize that while *VEGF*-TG (+) lungs had increased staining of vascular markers but the blood vessels looked disorganized which suggested leaky blood vessels, as noted by increased edema. However, in *VEGF*-TG */ NOS2*^−/−^ and *VEGF*-TG */ NOS3*^+/−^ mouse lungs the staining of these markers showed a well-arranged pattern of blood vessels (**Fig 3**), which correlated with less edema in these lungs (**Fig 5**). These data suggest the possibility that *VEGF*-TG (+) lungs have more microvessels as compared to WT, and this is reversed by NOS2 and NOS3 deletion. These studies demonstrate that VEGF induces angiogenesis and edema in the NB mice lungs via a mechanism that is, at least in part, NOS2 and NOS3 dependent.

### Effect of specific NOS isoform inhibition on lung injury markers (hemosiderin, nitrotyrosine and 8-OHdG) in the neonatal VEGF-TG (+) lung at PN7 in RA

**Fig 6:**
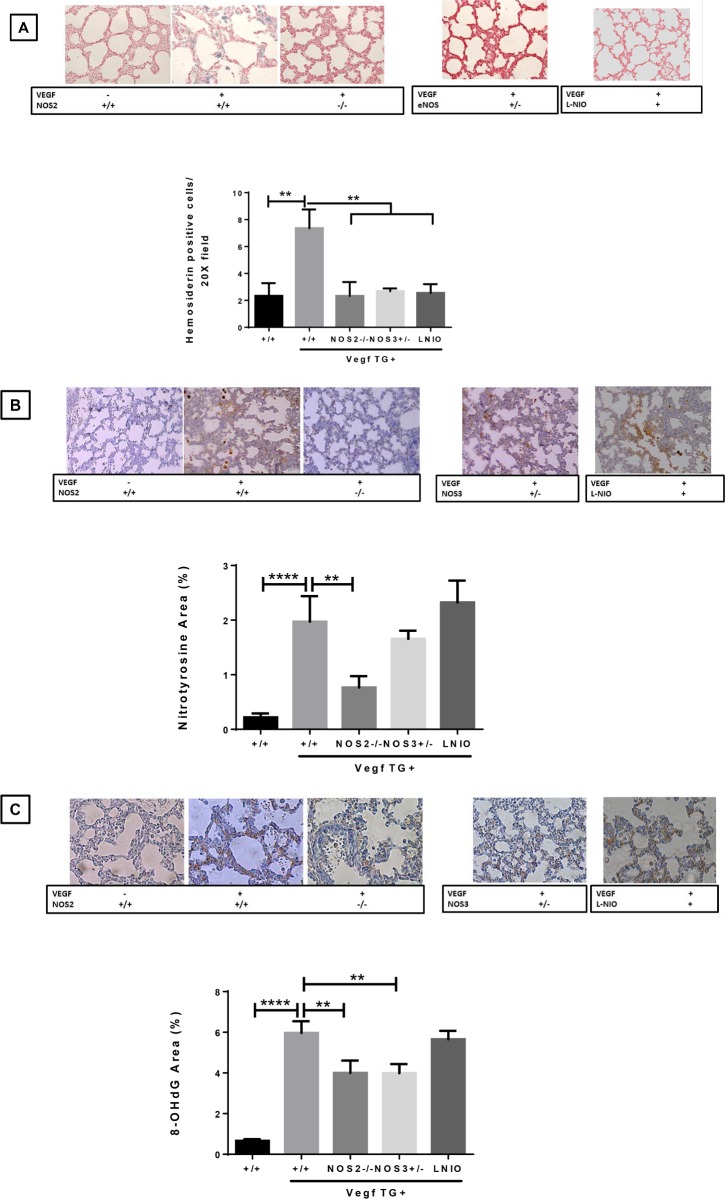
Role of NOS isoforms in VEGF overexpressed lung on oxidative stress. (**A**) PN7 lungs were removed from indicated groups, fixed, stained to allow identification of hemosiderin-laden macrophages. Hemosiderin-laden activated macrophages accumulate in VEGF TG+ PN7 mice lungs, which were significantly reduced in VEGF-TG/ NOS2^−/−^, VEGF-TG/ NOS3^+/−^ and NOS1 inhibited mice lungs. (**B**) Representative examples of nitrotyrosine staining and quantitation from lung of 7-day-old VEGF TG-, VEGF TG+, VEGF-TG/ NOS2^−/−^ and VEGF-TG/ NOS3^+/−^ mice exposed to DOX water from PN1-PN7. (**C**) Representative examples of staining and quantitation of lung 8-OHdG in VEGF TG+ PN7 newborn mice, which is significantly decreased in VEGF-TG+/ NOS2^−/−^ and VEGF-TG+/ NOS3^−/−^ lungs. All immunohistochemistry images were quantified by NIH image J software. Each bar represents the mean ± SEM for a minimum of 3 animals. **P ≤ 0.01 and ****P ≤ 0.0001, 2-way ANOVA followed by Tukey test. NOS: nitric oxide synthase; VEGF: vascular endothelial growth factor; TG-: transgene negative; TG+: transgene positive; PN: postnatal; DOX: doxycycline; 8-OHdG: 8-hydroxy-2'-deoxyguanosine.

Lung macrophages are loaded with Fe that are kept in an unreactive state by ferritin and hemosiderin. Hemosiderin, which results from incomplete lysosomal degradation of ferritin, binds lysosomal Fe even stronger than ferritin [14]. Activation of the VEGF transgene caused a significant increase in pleural and parenchymal hemorrhage and the accumulation of hemosiderin-laden macrophages, as previously reported [4]. Specific NOS isoforms played a significant role in these responses because VEGF-induced hemorrhage disappeared in *VEGF*-TG / *NOS2*^−/−^, *VEGF*-TG / *NOS3*^+/−^, and NOS1 inhibited lungs, with a marked diminution in hemosiderin staining (**Fig 6A**).

Nitrotyrosine and 8-OHdG staining were used as markers to measure oxidative stress in the lung. In the NB *VEGF*-TG (+) mouse model, the inhibition of NOS2 and NOS3 resulted in a marked decrease in VEGF induced nitrotyrosine- and 8-OHdG positive cells in PN7 lungs (**Fig 6B–6C**). NOS1 inhibition, in the presence of VEGF activation, did not appear to impact on these markers (**Fig 6B and 6C**).

Taken together, these data suggest that VEGF increased hemosiderin, nitrotyrosine and 8-OHdG in the developing mouse lung, and acted mostly via the NOS2 and NOS3-dependent pathways, at least in part, in augmenting lung injury.

Thus, in terms of contribution of the individual NOSs, our data would suggest that in the VEGF overexpressing neonatal lung at PN7 in RA, the effects on alveolar and vascular development and injury are primarily mediated by NOS2 and NOS3, but not NOS1.

### Effects of VEGF overexpression on lung phenotype, caspase 3 and cathepsins mRNA expression in neonatal lungs in the NB hyperoxia-induced model of BPD at PN14

Using NB Sprague-Dawley rats, it has been reported that human recombinant VEGF treatment improves alveolarization, despite resulting in transient pulmonary edema, during or after hyperoxia-exposure [15, 16]. In addition, adenoviral-vector mediated VEGF gene transfer using the same model also enhances angiogenesis and improves alveolar structure [17]. In striking contrast, we have previously reported that increased VEGF pulmonary levels concomitant with hyperoxia exposure is associated with increased mortality, lung oxidant injury and cell death in NB mice and increased initial (first 12h of life) VEGF levels are associated with human BPD [4].

Given the NB rat data that enhanced VEGF-NO signaling is important in improving the pulmonary phenotype of BPD [18–20], we sought to determine if enhancing VEGF pulmonary levels in the alveolar phase of lung development in RA, post-hyperoxic injury, would be beneficial in preserving or improving lung architecture, in NB mice.

**Fig 7:**
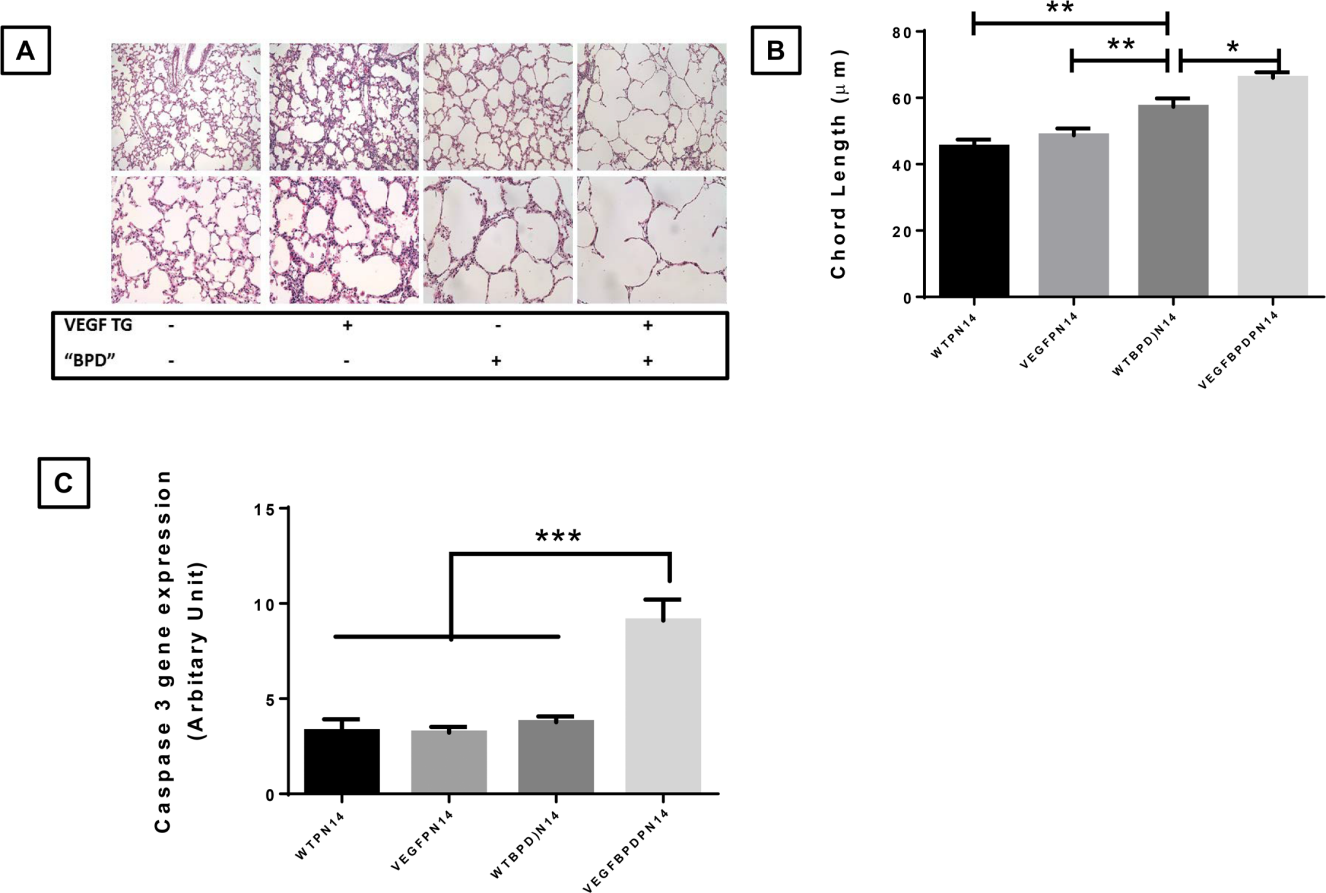
Effect of VEGF overexpression on pulmonary phenotype and caspase 3 mRNA expression in the BPD murine model. NB WT and VEGF TG+ mice were exposed to room air or hyperoxia to induce the murine model of BPD (hyperoxia from PN1-4; room air from PN5-PN14) and were killed on PN14. All received DOX water from PN day 5 to 14. (**A**) Representative microphotographs from H&E-stained lung sections showed alveolar size, as confirmed by chord length measurements (**B**), demonstrated the increased simplification in the NB VEGF TG+ mice as compared to VEGF TG- (WT) in hyperoxia-induced murine model of BPD. (**C**) The mRNA expression of caspase 3 was also increased in VEGF TG+ lung in NB BPD lungs. Each bar represents the mean ± SEM for a minimum of four animals. Each bar represents the mean ± SEM for a minimum of four animals. *P < 0.05, **P ≤ 0.01 and ***P ≤ 0.001, 2-way ANOVA followed by Tukey test. VEGF: vascular endothelial growth factor; BPD: bronchopulmonary dysplasia; NB: newborn; WT: wild type; NOS: nitric oxide synthase; TG+: transgene positive; TG-: transgene negative; PN: postnatal; DOX: doxycycline.

We [5, 9], and others [21], have reported that a mouse model of BPD mimics human BPD in pulmonary phenotype, and long-term physiologic consequences. NB WT mice subjected to hyperoxia from PN1-4, followed by recovery for next 10 days, have alveolar simplification characteristic of BPD (**Fig 7A**). However, the exposure of *VEGF*-TG (+) mouse pups to hyperoxia from PN1-4 followed by recovery in the presence of VEGF overexpression (DOX ON) for the next 10 days further worsened the pulmonary phenotype (**Fig 7A and 7B**). We confirmed that VEGF levels were appropriately increased in the *VEGF*-TG (+) mice lungs (**S3 Fig**).

In order to assess the mechanism of the worsening pulmonary pheonotype, we noted significantly increased lung caspase 3 gene expression in the *VEGF*-TG (+) BPD model as compared to controls (**Fig 7C**).

**Fig 8:**
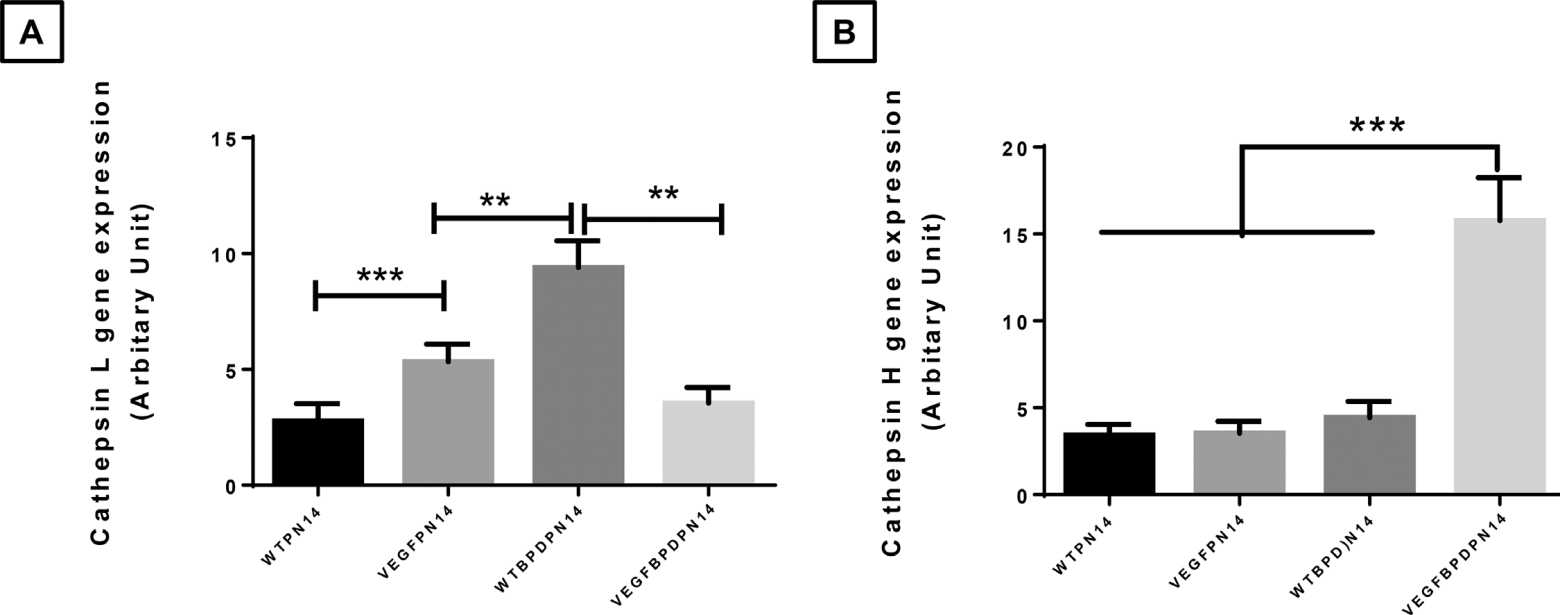
Effect of VEGF overexpression on cathepsins L and H mRNA expression in the BPD murine model. NB WT and VEGF TG+ mice were exposed to room air or hyperoxia to induce the murine model of BPD (hyperoxia from PN1-4; room air from PN5-PN14) and were killed on PN14. All received DOX water from PN day 5 to 14. (**A**) Cathepsin L mRNA expression in lung tissue was decreased in VEGF TG+ mice as compared to VEGF TG- mice, in the BPD mice model. (**B**) Cathepsin H mRNA expression in lung tissue was increased in VEGF TG+ mice as compared to VEGF TG- mice, in the BPD mice model. Each bar represents the mean ± SEM for a minimum of four animals. **P ≤ 0.01 and ***P ≤ 0.001, 2-way ANOVA followed by Tukey test. VEGF: vascular endothelial growth factor; BPD: bronchopulmonary dysplasia; NB: newborn; WT: wild type; NOS: nitric oxide synthase; TG+: transgene positive; TG-: transgene negative; PN: postnatal; DOX: doxycycline.

We [22] and several other authors have reported a role of cathepsins in lung development and hyperoxia induced lung injury [23, 24]. Hence, we also evaluated mRNA expression of all cathepsins but only found noticeable differences in cathepsin L and H expression. Cathepsin L was increased in NB *VEGF*-TG (+) RA and WT BPD lungs but was down-regulated to normal levels in the *VEGF*-TG (+) BPD lungs (**Fig 8A**). In contrast, cathepsin H mRNA was increased in *VEGF*-TG (+) BPD as compared to WT BPD lungs (**Fig 8B**). Increased levels and activity of cathepsin H have been detected in the baboon model of BPD [25]. During lung development Cathepsin H regulates signaling by the fibroblast growth factor 10 (Fgf10) which is critical for induction of a gene network that controls proliferation, differentiation, and branching of the epithelial tubules [26].

Taken together, our data would suggest that in NB mice, provision of increased VEGF in the alveolar phase of lung development (PN5-14) in RA, following hyperoxia exposure in the saccular stage of lung development (PN1-4), worsens the BPD pulmonary phenotype. Based on our gene expression data, increased cell death and altered proteases could be responsible, at least in part, for this finding.

## Discussion

The findings of this study demonstrate that NOS2 and NOS3 deficiency, but not NOS1 inhibition, significantly attenuates VEGF-induced alveolar simplification, vascular development and lung injury in NB mice in RA. Through generation of double genetically altered *VEGF*-TG/ *NOS2*^−/−^ and *VEGF*-TG/ *NOS3*^+/−^ mice, we show that these 2 NOS isoforms are primarily responsible for modulating VEGF-induced responses in the developing lung. We suggest that differential cell proliferation rates could be responsible for the restoration of alveolar architecture. Also, we indicate specific molecular markers of vascular development and lung injury are altered in response to NOS deletion in the *VEGF*-TG (+) developing lung. In addition, we note that pulmonary expression of an active VEGF165 isoform, in the post-hyperoxic phase (i.e. in RA), worsened the pulmonary phenotype, in the hyperoxia-induced mouse model of BPD, exemplified by an increase in alveolar simplification. We suggest that this result could be mediated, at least in part, by increased cell death (increased caspase 3 expression) and alterations in the expression of proteases (specifically, an increase in cathepsin H, but decreased cathepsin L expression).

Our data showed that the lack of NOS2 and NOS3 confers protection against VEGF-induced pathologic lung vascular development, morphology and injury. Consistent with our results, a recent study proves that osteopontin confers protection against hyperoxia-induced lung injury by inhibiting NOS2 and NOS3 [27]. Another study elucidated the role of three NOS isoforms in hyperoxia induced lung injury and reported protective effects of NOS3 but deleterious roles of NOS1 and NOS2 using different lung injury models [28]. Several other studies also support our study that NOS2 and NOS3 inhibition is beneficial in lung injury [29, 30]. NO production maintains lung vascular growth and development *in utero* [31]. We speculate that VEGF-NOS is essential for pulmonary angiogenesis in the lung exposed to increased oxygen tension and that impaired NO production may contribute to the abnormalities of angiogenesis. However, in the VEGF overexpression room air model excess of VEGF leads to increased but dysregulated angiogenesis, which is restored towards a more normal pattern by NOS2 and NOS3 deletion. While we were unable to show a major role of NOS1 in VEGF-mediated effects in our NB VEGF TG mice lungs, we can confirm that the NOS1 inhibitor (L-NIO) dose used was sufficient to decrease the expression of NOS1 (**S1 Fig**).

Other studies have shown similar results in that NO augments pulmonary angiogenesis *in vitro* or *in vivo* via modulation of angiogenic factors [31–33]. This study is the first to demonstrate the detrimental effects of upregulated VEGF activity on angiogenesis in the developing lung, at least in part, is the result of enhanced NOS activity, as evidenced by a more normal pattern of angiogenesis in NOS2 and NOS3 null/heterozygous mice. Our experiments show that ablation NOS2 or NOS3 production can normalize/decrease angiogenesis, oxidative stress and vascular permeability in VEGF TG mice as evident by vascular marker staining, 8-OHdG staining and BAL protein levels. This is in accordance with what has been previously reported, as endothelial NO is clearly pro-angiogenic: NO has been shown to promote increased vascular permeability, endothelial cell migration and proliferation [33].

Administration of supplemental oxygen can cause lung injury when administered at high concentrations. Previous studies from our laboratory demonstrated that VEGF-NOS pathway in the murine lung modulate the consequences of exposure to 100% oxygen [2, 4, 34]. Our present studies demonstrate that NOS isoforms plays an important role in this lung cytoprotective response because, in the absence of NOS2 and NOS3, the VEGF-induced lung injury was markedly diminished. When viewed in combination, these studies demonstrate that VEGF induces different isoforms of NOS in the neonatal murine lung and this pathway plays an important role in mediating the cytoprotective and oxidative stress pathways.

The prior animal literature utilizing VEGF/hypoxia-inducible factor-1α supplementation concurrently or post-hyperoxia [15–17, 35] would have predicted improved alveolarization in our model. However, NB rats [15–17] or preterm baboons [35] were used in these studies, which could possibly account for the different results. In addition, the timing and duration of VEGF activation which would impact on the VEGF levels in the lung could also be contributing to our present and previously reported [4] results. Investigators have reported diminished VEGF and VEGF-R2 expression in lung specimens from human and baboon BPD [36, 37], and detrimental effects of VEGF inhibition/receptor deletion on lung structure [38, 39]. While, as before, the different animal models could explain this discrepancy, it is important to note that the kinetics of VEGF release/production impacts on the timing of measurement [40], even in preterm neonates [4]. Thus, it is apparent that extrapolation of VEGF data from animals to humans needs to be done with caution [40]. In addition, a fine balance of timing as well as dose and duration of VEGF administration is required for interpreting the various beneficial or detrimental effects of VEGF in developing lungs exposed to hyperoxia.

In summary, our studies demonstrate that, in contrast to the adult lung, VEGF is a potent stimulator of all 3 NOSs: NOS1, NOS2 and NOS3 in the NB mouse lung. In room air, we report that VEGF-induced lung simplification (increased chord length), cell proliferation, vascular development, edema, pulmonary oxidative stress and injury is NOS2 and NOS3 dependent, with limited, if any, role of NOS1. In addition, VEGF activation worsened alveolar simplification in the mouse model of BPD in the NB. Our data show that there is a significant differential regulation in the NOS-mediated effects of VEGF overexpression in the developing mouse lung.

## Supporting Information

S1 Fig Inhibition of NOS1. Specific inhibitor Vinyl-L-NIO hydrochloride (LNIO) was used in WT and VEGF TG+ mice. The animals were killed on PN7 and lung tissue were used for western blot analysis. VEGF: vascular endothelial growth factor; WT: wild type; NOS1: nitric oxide synthase1; TG+: transgene positive.
(TIF)

S2 Fig Role of NOS isoforms in VEGF overexpressed lung on surfactant protein expression. (**A**) SP-A, SP-B, SP-C and SP-D mRNA, with β-actin were detected by semi-quantitative PCR in L-NIO (NOS1 inhibitor) treated VEGF TG mice. (**B–D)** NB VEGF TG-, VEGF TG+, VEGF-TG/ NOS2^−/−^ and VEGF-TG/ NOS3^+/−^ mice were sacrificed at PN7. All received DOX water from PN day 1 to 7. SP-A, SP-B, SP-C and SP-D proteins and mRNA, with β-actin as controls, were detected by western blotting and semi-quantitative PCR. The figure is representative of n = 3 mice per group. NOS: nitric oxide synthase; VEGF: vascular endothelial growth factor; NB: newborn; TG-: transgene negative; TG+: transgene positive; PN: postnatal; DOX: doxycycline; SP-A: surfactant protein A, SP-B: surfactant protein B, SP-C: surfactant protein C, SP-D: surfactant protein D.
(TIF)

S3 Fig VEGF levels in the PN14 room air and BPD murine model. **(A)** NB WT and VEGF TG+ mice were exposed to room air or hyperoxia to induce the murine model of BPD (hyperoxia from PN1-4; room air from PN5-PN14) and were killed on PN14. All received DOX water for VEGF overexpression from PN day 5 to 14. There were no noticeable differences in VEGF levels in room air and BPD animals after DOX administration. Each bar represents the mean ± SEM for a minimum of four animals. ***P ≤ 0.001, 2-way ANOVA followed by Tukey test. VEGF: vascular endothelial growth factor; PN: postnatal; BPD: Bronchopulmonary dysplasia; NB: newborn; WT: wild type; TG-: transgene negative; TG+: transgene positive; DOX: doxycycline.
(TIF)
