# Supplementary figures and images for: Role of Nitric Oxide Isoforms in Vascular and Alveolar Development and Lung Injury in Vascular Endothelial Growth Factor Overexpressing Neonatal Mice Lungs
Source: PLoS One. 2016 Jan 22;11(1):e0147588. doi: 10.1371/journal.pone.0147588 (PMC4723240; doi:10.1371/journal.pone.0147588)

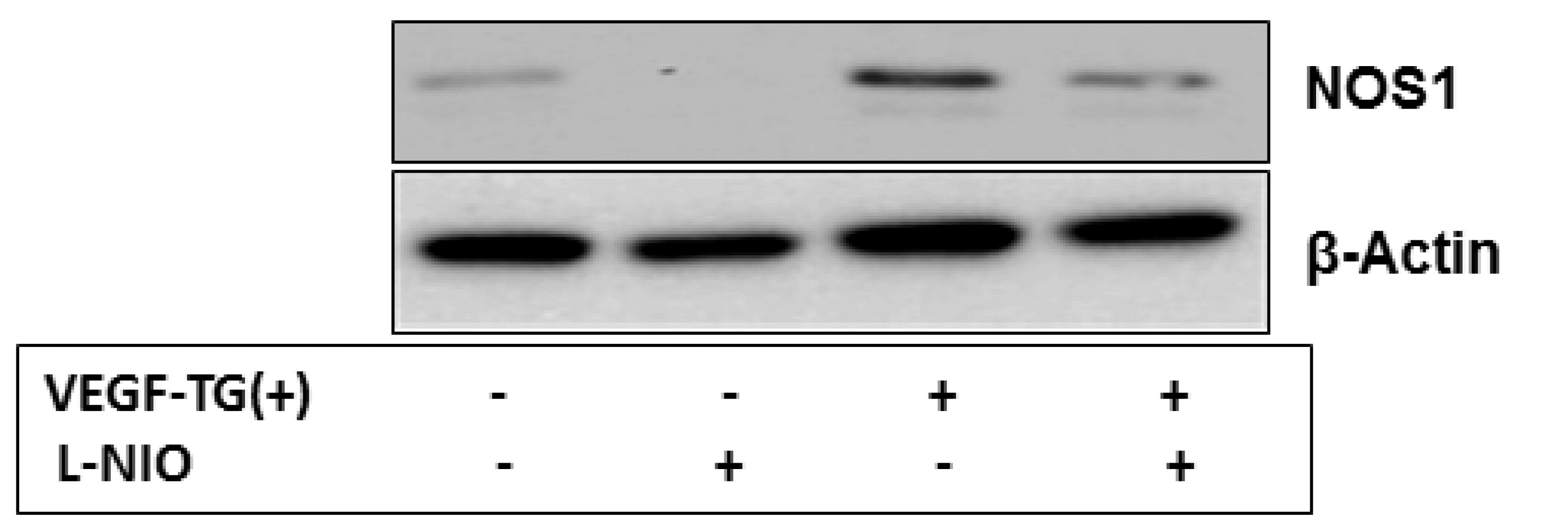

Supplement: S1 Fig — Specific inhibitor Vinyl-L-NIO hydrochloride (LNIO) was used in WT and VEGF TG+ mice. The animals were killed on PN7 and lung tissue were used for western blot analysis. VEGF: vascular endothelial growth factor; WT: wild type; NOS1: nitric oxide synthase1; TG+: transgene positive. (TIF) [file pone.0147588.s001.tif]

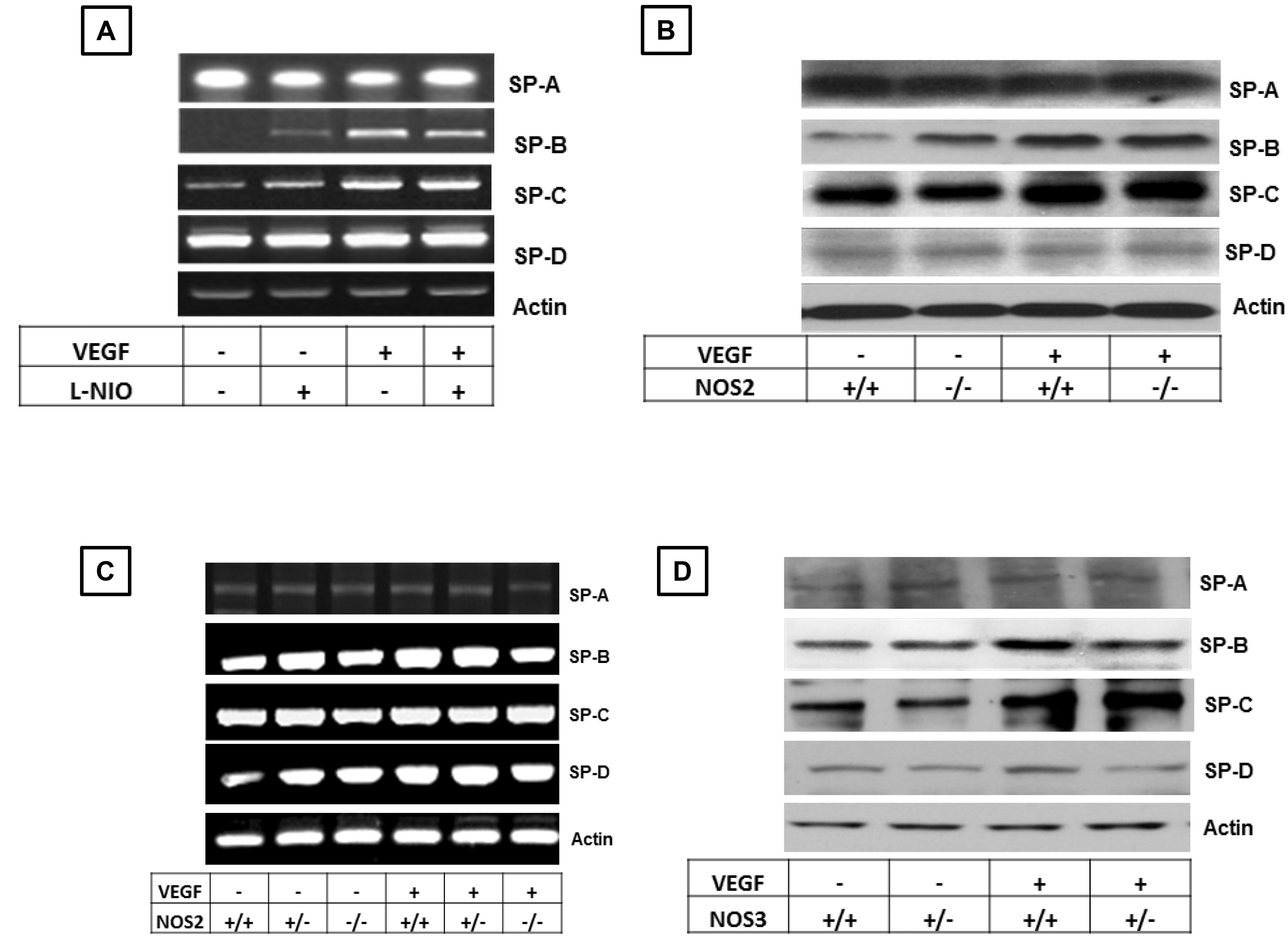

Supplement: S2 Fig — (A) SP-A, SP-B, SP-C and SP-D mRNA, with β-actin were detected by semi-quantitative PCR in L-NIO (NOS1 inhibitor) treated VEGF TG mice. (B–D) NB VEGF TG-, VEGF TG+, VEGF-TG/ NOS2−/− and VEGF-TG/ NOS3+/− mice were sacrificed at PN7. All received DOX water from PN day 1 to 7. SP-A, SP-B, SP-C and SP-D proteins and mRNA, with β-actin as controls, were detected by western blotting and semi-quantitative PCR. The figure is representative of n = 3 mice per group. NOS: nitric oxide synthase; VEGF: vascular endothelial growth factor; NB: newborn; TG-: transgene negative; TG+: transgene positive; PN: postnatal; DOX: doxycycline; SP-A: surfactant protein A, SP-B: surfactant protein B, SP-C: surfactant protein C, SP-D: surfactant protein D. (TIF) [file pone.0147588.s002.tif]

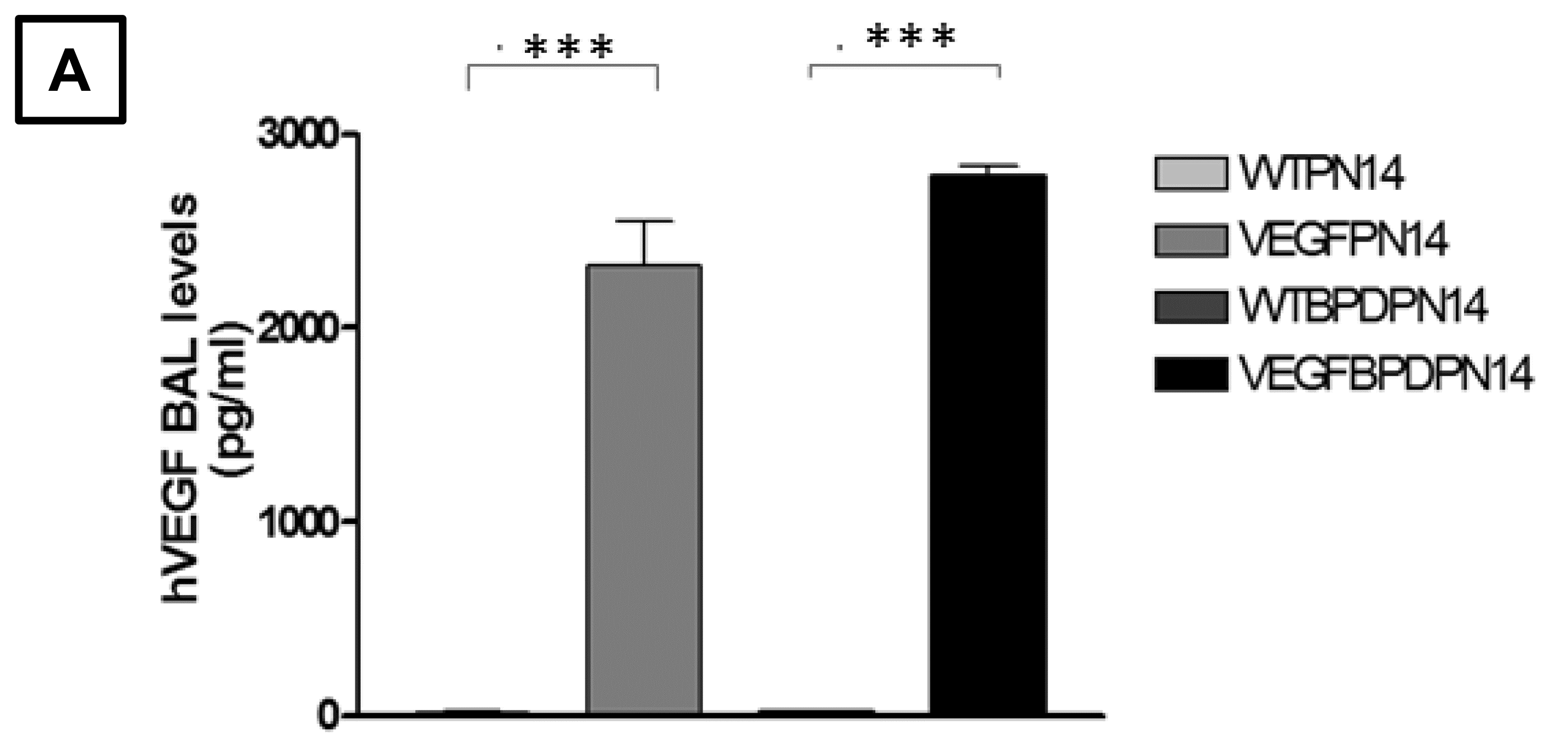

Supplement: S3 Fig — (A) NB WT and VEGF TG+ mice were exposed to room air or hyperoxia to induce the murine model of BPD (hyperoxia from PN1-4; room air from PN5-PN14) and were killed on PN14. All received DOX water for VEGF overexpression from PN day 5 to 14. There were no noticeable differences in VEGF levels in room air and BPD animals after DOX administration. Each bar represents the mean ± SEM for a minimum of four animals. ***P ≤ 0.001, 2-way ANOVA followed by Tukey test. VEGF: vascular endothelial growth factor; PN: postnatal; BPD: Bronchopulmonary dysplasia; NB: newborn; WT: wild type; TG-: transgene negative; TG+: transgene positive; DOX: doxycycline. (TIF) [file pone.0147588.s003.tif]
